# Hepatitis C virus antibody seropositivity is associated with albuminuria but not peripheral artery disease in patients with type 2 diabetes

**DOI:** 10.1038/s41598-024-55352-7

**Published:** 2024-02-26

**Authors:** Yu-Cheng Cheng, Teng-Yu Lee, Yu-Hsuan Li, Chin-Li Lu, Hsiu-Chen Liu, Meei Ling Sheu, I-Te Lee

**Affiliations:** 1 Division of Endocrinology and Metabolism, Department of Internal Medicine, Taichung Veterans General Hospital, Taichung, 40705 Taiwan; 2 School of Medicine, National Yang Ming Chiao Tung University, Taipei, 11221 Taiwan; 3 Institute of Biomedical Sciences, National Chung Hsing University, Taichung, 40227 Taiwan; 4 Division of Gastroenterology and Hepatology, Department of Internal Medicine, Taichung Veterans General Hospital, Taichung, 40705 Taiwan; 5 School of Medicine, Chung Shan Medical University, Taichung, 40201 Taiwan; 6 Department of Computer Science and Information Engineering, National Taiwan University, Taipei, 10617 Taiwan; 7 Graduate Institute of Food Safety, College of Agriculture and Natural Resources, National Chung Hsing University, Taichung, 40227 Taiwan; 8 Department of Nursing, Taichung Veterans General Hospital, Taichung, 40705 Taiwan

**Keywords:** Type 2 diabetes, Hepatitis C

## Abstract

Hepatitis C virus (HCV) infection is prevalent in patients with type 2 diabetes mellitus (DM). We aimed to investigate whether HCV antibody (Ab) seropositivity is associated with diabetic micro- and macro-vascular diseases. In this hospital-based cross-sectional study, we retrospectively collected data from patients who participated in the diabetes pay-for-performance program and underwent HCV Ab screening in the annual comprehensive assessment between January 2021 and March 2022. We examined the relationships of HCV Ab seropositivity with the spot urinary albumin-to-creatinine ratio (UACR) and ankle-brachial index (ABI) in patients aged ≥ 50 years with type 2 DM. A total of 1758 patients were enrolled, and 85 (4.83%) of the enrolled patients had HCV Ab seropositivity. Multivariable regression analyses revealed that albuminuria showed a dose-dependent association with HCV Ab seropositivity (UACR [30–299 mg/g]: odds ratio [OR] = 1.463, 95% confidence interval [CI] 0.872‒2.456); UACR [≥ 300 mg/g]: OR = 2.300, 95% CI 1.160‒4.562; *P* for trend = 0.015) when compared with normal albuminuria (UACR < 30 mg/g). However, the proportion of patients with peripheral arterial disease, defined as an ABI ≤ 0.9, was not significantly different between the groups with and without HCV Ab seropositivity (3.5% vs. 3.9%, *P* = 0.999). In conclusion, severely increased albuminuria, but not the ABI, showed a significant association with HCV Ab seropositivity in patients aged ≥ 50 years with type 2 DM.

## Introduction

Hepatitis C virus (HCV) infection is a major cause of chronic liver disease, liver cirrhosis, and hepatocellular carcinoma^1,2^. The global prevalence of HCV antibody (HCV Ab) seropositivity increased from 2.3% in 1990 to 2.8% in 2005^3^. The prevalence of HCV viremia was also reported to be approximately 1%^4^. Chronic hepatitis C infection and its sequelae place a global burden on the health care system and the economy^1^. In addition to hepatic sequelae, HCV causes several extrahepatic diseases that contribute significantly to the burden of this disease^5^. Type 2 diabetes mellitus (DM) is one of the most frequent and best characterized HCV-associated extrahepatic manifestations^6^. A higher risk of type 2 DM was reported in people with HCV infection than in those without HCV infection^7^, and the rate of HCV infection was also higher in people with type 2 DM than in those without type 2 DM^8^. Type 2 DM can cause several micro- and macro-vascular complications, such as nephropathy and cardiovascular disease (CVD)^9^, and HCV infection has also been reported to increase the risk of CVD and chronic kidney disease (CKD)^10,11^. Moreover, HCV treatment could reduce the risk of CVD and end-stage renal disease (ESRD) in patients with DM^12^. Therefore, early screening of HCV infection is an important concern for patients with DM.

Peripheral arterial disease (PAD) is a common macrovascular complication in patients with type 2 DM^13^. The ankle-brachial index (ABI) is a convenient tool to screen for PAD and is recommended for all patients with DM aged older than 50 years^14^. HCV infection increases the risk of developing PAD^15^. However, studies exploring the association between PAD based on ABI assessment and HCV infection in patients with DM are limited. On the other hand, albuminuria is a risk factor for CVD, diabetic kidney disease progression and all-cause mortality in patients with DM^16,17^. The spot urinary albumin-to-creatinine ratio (UACR) is widely recommended as a marker for quantifying proteinuria in patients with DM^18^. Increased albuminuria has been reported in patients with HCV infection^19–21^. However, to the best of our knowledge, studies exploring the association between the UACR and HCV infection have not focused on patients with type 2 DM.

Taiwan is a hyperendemic area for HCV infection^22^. The prevalence of HCV Ab seropositivity was reported to be 3.28% in the general population^23^, and to be 7.8% in the population with type 2 DM aged between 40 and 65 years^24^. To identify HCV infection is essential for the HCV elimination policy in Taiwan. According to the Taiwan hepatitis C policy guidelines^25^, people aged ≥ 45 years should be screened for HCV Ab. Furthermore, based on the recommendations of the diabetes pay-for-performance (P4P) program, an annual comprehensive assessment including the UACR and PAD should be performed for all patients with DM. We hypothesized that albuminuria and PAD would be associated with HCV infection. Therefore, we conducted a cross-sectional study with the aim of examining the prevalence of HCV Ab seropositivity in patients with type 2 DM categorized by the UACR or ABI.

## Results

**Figure 1:**
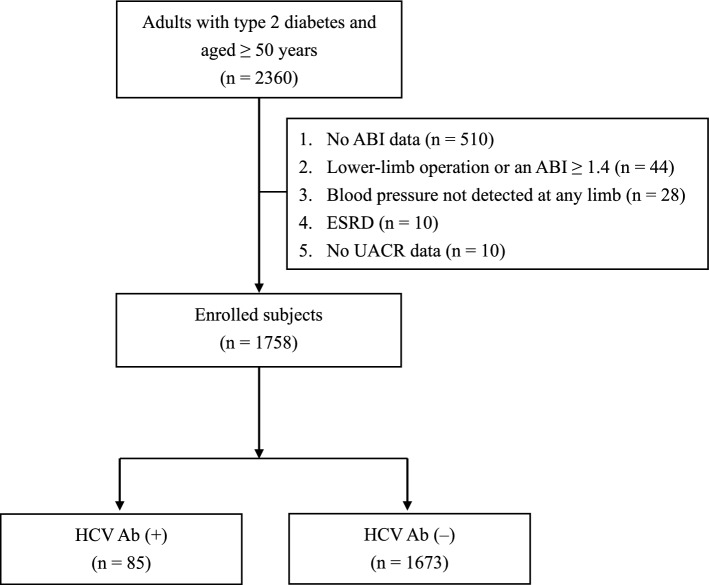
Flow diagram of the enrollment of study subjects. *ABI* ankle-brachial index, *ESRD* end-stage renal disease, *HCV Ab* hepatitis C virus antibody, *UACR* urinary albumin-to-creatinine ratio.

A total of 1758 patients with type 2 DM were enrolled in the study (Fig. 1), and their characteristics are shown in Table 1. There were 85 (4.83%) patients with HCV Ab seropositivity. Compared with the group without HCV Ab seropositivity, the group with HCV Ab seropositivity was older (69 ± 8 vs. 66 ± 9 years, *P* = 0.002) and had a higher systolic blood pressure (BP) (139 ± 17 vs. 133 ± 18 mm Hg, *P* = 0.004), a lower estimated glomerular filtration rate (eGFR) (68 ± 20 vs. 73 ± 22 mL/min/1.73 m^2^, *P* = 0.034), a higher UACR (274 ± 611 vs. 123 ± 433 mg/g, *P* = 0.002), a lower proportion of males (41.2% vs. 57.4%, *P* = 0.005), and a higher proportion of patients receiving insulin therapy (29.4% vs. 19.4%, *P* = 0.035). There was no significant difference in the proportion of patients with hypertension, the proportion of patients with CVD, diabetes duration, body mass index (BMI), diastolic BP, fasting glucose, hemoglobin A1c (HbA1c), total cholesterol, low density lipoprotein (LDL) cholesterol, high density lipoprotein (HDL) cholesterol, triglycerides, alanine aminotransferase (ALT), ABI, the proportion of patients with antiplatelet use, the proportion of patients with statin use, the proportion of patients with antihypertensive drug use, the proportion of patients with angiotensin-converting enzyme inhibitor or angiotensin II receptor antagonist use, or the proportion of patients with oral antihyperglycemic drug use between the groups with and without HCV Ab seropositivity.

**Table 1:** The characteristics of enrolled subjects with and without HCV Ab seropositivity.

	HCV Ab ( +) N = 85	HCV Ab (‒) N = 1673	*P*
Age (years)	69 ± 8	66 ± 9	0.002
Male, n (%)	35 (41.2)	960 (57.4)	0.005
Hypertension, n (%)	69 (81.2)	1312 (78.4)	0.640
CVD history, n (%)	56 (65.9)	1064 (63.6)	0.755
Diabetes duration (years)	12 ± 8	13 ± 8	0.712
BMI (kg/m^2^)	25.9 ± 4.4	25.9 ± 4.1	0.954
Systolic BP (mm Hg)	139 ± 17	133 ± 18	0.004
Diastolic BP (mm Hg)	75 ± 10	75 ± 11	0.822
Fasting glucose (mmol/L)	7.5 ± 2.2	7.4 ± 2.0	0.677
HbA1c (%)	7.0 ± 1.0	7.0 ± 1.1	0.959
Total cholesterol (mmol/L)	3.9 ± 0.7	3.9 ± 0.8	0.561
LDL cholesterol (mmol/L)	2.1 ± 0.6	2.1 ± 0.7	0.866
HDL cholesterol (mmol/L)	1.4 ± 0.4	1.3 ± 0.4	0.236
Triglycerides (mmol/L)	2.8 ± 1.5	3.2 ± 2.5	0.218
ALT (U/L)	24 ± 17	25 ± 19	0.797
eGFR (mL/min/1.73 m^2^)	68 ± 20	73 ± 22	0.034
UACR (mg/g)	274 ± 611	123 ± 433	0.002
ABI	1.1 ± 0.1	1.1 ± 0.1	0.461
Use of antiplatelet drugs, n (%)	19 (22.4)	431 (25.8)	0.565
Use of statins, n (%)	64 (75.3)	1364 (81.5)	0.196
Use of antihypertensive drugs, n (%)	49 (57.6)	842 (50.3)	0.228
Use of ACE inhibitors or ARBs, n (%)	33 (38.8)	592 (35.4)	0.596
Insulin therapy, n (%)	25 (29.4)	325 (19.4)	0.035
Oral antihyperglycemic drugs, n (%)	78 (91.8)	1596 (95.4)	0.204
Insulin secretagogues, n (%)	24 (28.2)	528 (31.6)	0.600
Metformin, n (%)	29 (34.1)	634 (37.9)	0.558
DPP4 inhibitors, n (%)	44 (51.8)	844 (50.4)	0.900
SGLT2 inhibitors, n (%)	26 (30.6)	527 (31.5)	0.955
Thiazolidinediones, n (%)	26 (30.6)	593 (35.4)	0.425
α-Glucosidase inhibitors, n (%)	8 (9.4)	82 (4.9)	0.112

*ABI* ankle-brachial index, *ACE* angiotensin-converting enzyme, *ALT* alanine aminotransferase, *ARB* angiotensin II receptor antagonist, *BMI* body mass index, *BP* blood pressure, *CVD* cardiovascular disease, *DPP4* dipeptidyl peptidase-4, *eGFR* estimated glomerular filtration rate, *HbA1c* glycated hemoglobin, *HDL* high-density lipoprotein, *HCV Ab* anti-hepatitis C virus antibody, *LDL* low-density lipoprotein, *SGLT2* sodium-glucose cotransporter 2, *UACR* urinary albumin-to-creatinine ratio.

**Figure 2:**
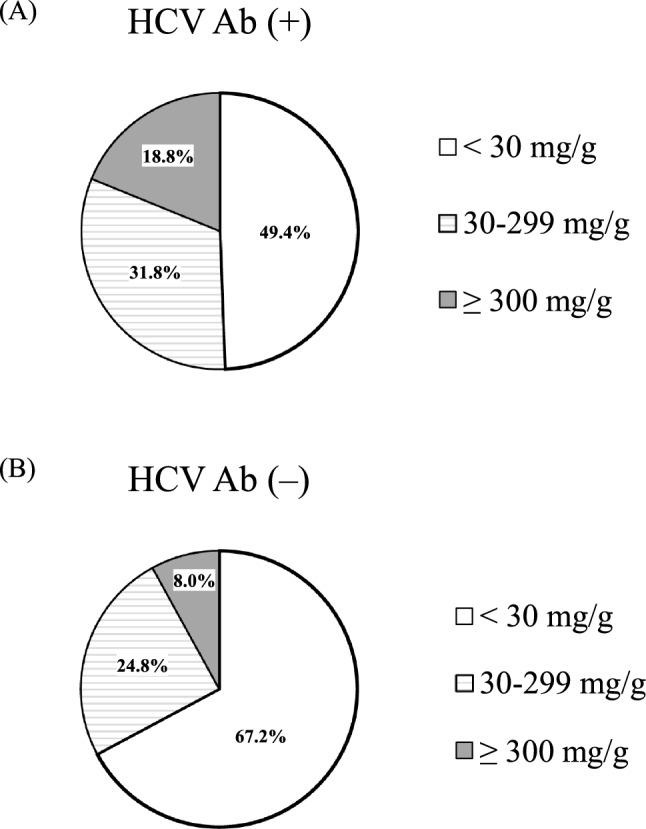
The prevalence of subjects in each UACR (mg/g) category between the groups with HCV Ab seropositivity (**A**) and without HCV Ab seropositivity (**B**). *HCV Ab* hepatitis C virus antibody, *UACR* urinary albumin-to-creatinine ratio.

The proportions of patients in different UACR categories between the groups with and without HCV Ab seropositivity are shown in Fig. 2. Patients with HCV Ab seropositivity had an increasing ratio trend in the severity of albuminuria compared to those without HCV Ab seropositivity; i.e., from normal albuminuria (49.4% vs. 67.2%) and moderately increased albuminuria (24.8% vs. 31.8%) to severely increased albuminuria (18.8% vs. 8.0%, *P* < 0.001). However, there was no significant difference in the proportion of patients with PAD between the groups with and without HCV Ab seropositivity (3.5% vs. 3.9%, *P* = 0.999).

To assess the potential risk factors for severely increased albuminuria, univariate regression analyses were performed (Table 2). Patients aged ≥ 65 years had a higher risk of severely increased albuminuria (odds ratio [OR] = 1.539, 95% confidence interval [CI] 1.082–2.188, *P* = 0.016) than subjects aged < 65 years; patients with hypertension had a higher risk of severely increased albuminuria (OR = 14.851, 95% CI 4.707–46.851, *P* < 0.001) than those without hypertension; and patients with a CVD history had a higher risk of severely increased albuminuria (OR = 3.243, 95% CI 2.072–5.076, *P* < 0.001) than those without a CVD history. Patients with a diabetes duration ≥ 10 years had a higher risk of severely increased albuminuria (OR = 2.078, 95% CI 1.420–3.043, *P* < 0.001) than those with a diabetes duration < 10 years. Patients with a BMI ≥ 25 kg/m^2^ had a higher risk of severely increased albuminuria (OR = 1.486, 95% CI 1.049–2.103, *P* = 0.026) than those with a BMI < 25 kg/m^2^; patients with a systolic BP ≥ 140 mm Hg had a higher risk of severely increased albuminuria (OR = 3.340, 95% CI 2.368‒4.711, *P* < 0.001) than those with a systolic B*P* < 140 mm Hg; and patients with a diastolic BP ≥ 90 mm Hg had a higher risk of severely increased albuminuria (OR = 3.360, 95% CI 2.203‒5.127, *P* < 0.001) than those with a diastolic B*P* < 90 mm Hg. An HbA1c level ≥ 7% was associated with a higher risk of severely increased albuminuria (OR = 2.149, 95% CI 1.523‒3.031, *P* < 0.001) than an HbA1c level < 7%; an LDL cholesterol level ≥ 2.59 mmol/L was associated with a higher risk of severely increased albuminuria (OR = 1.588, 95% CI1.086‒2.321, *P* = 0.017) than an LDL cholesterol level < 2.59 mmol/L; a low HDL cholesterol level was associated with a higher risk of severely increased albuminuria (OR = 1.873, 95% CI1.337‒2.625, *P* < 0.001) than a high HDL cholesterol level; and a triglyceride level ≥ 1.7 mmol/L was associated with a higher risk of severely increased albuminuria (OR = 4.918, 95% CI2.280‒10.608, *P* < 0.001) than a triglyceride level < 1.7 mmol/L. Patients with CKD had a higher risk of severely increased albuminuria (OR = 5.478, 95% CI 3.862‒7.771, *P* < 0.001) than those without CKD. Patients with an ABI ≤ 0.9 had a higher risk of severely increased albuminuria (OR = 2.962, 95% CI 1.604‒5.471, *P* < 0.001) than those with an ABI > 0.9. Patients who used antihypertensive drugs had a higher risk of severely increased albuminuria (OR = 2.359, 95% CI 1.645‒3.382, *P* < 0.001) than those who did not use antihypertensive drugs, and patients who received insulin therapy had a higher risk of severely increased albuminuria (OR = 4.023, 95% CI 2.846‒5.685, *P* < 0.001) than those who did not receive insulin therapy.

**Table 2:** The odds ratios (ORs) for severely increased albuminuria (UACR ≥ 300 mg/g) by assessed risk factors using univariate regression analyses.

Potential risk factors	UACR		OR (95% CI)	*P*
	≥ 300 mg/g n = 150	< 300 mg/g n = 1608		
	n (%)	n (%)		
Age ≥ 65 years	99 (66.0)	897 (55.8)	1.539 (1.082, 2.188)	0.016
Male	87 (58.0)	908 (56.5)	1.065 (0.759, 1.494)	0.717
Hypertension	147 (98.0)	1234 (76.7)	14.851 (4.707, 46.851)	< 0.001
CVD history	126 (84.0)	994 (61.8)	3.243 (2.072, 5.076)	< 0.001
Diabetes duration ≥ 10 years	112 (74.7%)	943 (58.6%)	2.078 (1.420, 3.043)	< 0.001
BMI ≥ 25 kg/m^2^	96 (64.0)	876 (54.5)	1.486 (1.049, 2.103)	0.026
Systolic BP ≥ 140 mm Hg	91 (60.7)	508 (31.6)	3.340 (2.368, 4.711)	< 0.001
Diastolic BP ≥ 90 mm Hg	34 (22.7)	129 (8.0)	3.360 (2.203, 5.127)	< 0.001
Fasting glucose ≥ 7.2 mmol/L	83 (55.3)	756 (47.0)	1.396 (0.997, 1.955)	0.052
HbA1c ≥ 7%	93 (62.0)	694 (43.2)	2.149 (1.523, 3.031)	< 0.001
Total cholesterol ≥ 4.14 mmol/L	63 (42.0)	567 (35.3)	1.330 (0.946, 1.868)	0.101
LDL cholesterol ≥ 2.59 mmol/L	41 (27.3)	308 (19.2)	1.588 (1.086, 2.321)	0.017
Low HDL cholesterol*	70 (46.7)	512 (31.8)	1.873 (1.337, 2.625)	< 0.001
Triglycerides ≥ 1.7 mmol/L	143 (95.3)	1296 (80.6)	4.918 (2.280, 10.608)	< 0.001
ALT ≥ 20 U/L	68 (45.3)	787 (49.0)	0.863 (0.617, 1.208)	0.390
eGFR < 60 mL/min/1.73 m^2^	93 (62.0)	369 (22.9)	5.478 (3.862, 7.771)	< 0.001
ABI ≤ 0.9	14 (9.3)	54 (3.4)	2.962 (1.604, 5.471)	< 0.001
Use of antiplatelet drugs	48 (32.0)	402 (25.0)	1.412 (0.984, 2.026)	0.061
Use of statins, n (%)	115 (76.7)	1313 (81.7)	0.738 (0.495, 1.100)	0.136
Use of antihypertensive drugs	104 (69.3)	787 (48.9)	2.359 (1.645, 3.382)	< 0.001
Use of ACE inhibitors or ARBs	70 (46.7)	555 (34.5)	1.660 (1.185, 2.325)	0.003
Use of insulin therapy	69 (46.0)	281 (17.5)	4.023 (2.846, 5.685)	< 0.001
Use of oral antihyperglycemic drugs	140 (93.3)	1534 (95.4)	0.675 (0.341, 1.337)	0.260
Insulin secretagogues	54 (36.0)	498 (31.0)	1.254 (0.884, 1.779)	0.205
Metformin	47 (31.3)	616 (38.3)	0.735 (0.513, 1.053)	0.093
DPP4 inhibitors	81 (54.0)	807 (50.2)	1.165 (0.833, 1.630)	0.372
SGLT2 inhibitors	50 (33.3)	503 (31.3)	1.098 (0.770, 1.567)	0.605
Thiazolidinediones	58 (38.7)	561 (34.9)	1.177 (0.834, 1.660)	0.355
α-Glucosidase inhibitors	5 (3.3)	85 (5.3)	0.618 (0.247, 1.547)	0.304

*ABI* ankle-brachial index, *ACE* angiotensin-converting enzyme, *ALT* alanine aminotransferase, *ARB* angiotensin II receptor antagonist, *BMI* body mass index, *BP* blood pressure, *CI* confidence interval, *CVD* cardiovascular disease, *DPP4* dipeptidyl peptidase-4, *eGFR* estimated glomerular filtration rate, *HbA1c* glycated hemoglobin, *HDL* high-density lipoprotein, *LDL* low-density lipoprotein, *OR* odds ratio, *SGLT2* sodium-glucose cotransporter 2, *UACR* urinary albumin-to-creatinine ratio.

*HDL cholesterol levels < 40 mg/dl (1.0 mmol/L) in men or < 50 mg/dl (1.3 mmol/L) in women.

To assess the association between albuminuria and the presence of HCV Ab seropositivity, we selected age, sex, and other potential confounding risk factors that were significantly associated with both HCV Ab seropositivity (in Table 1) and severely increased albuminuria (in Table 2) for the multivariable logistic regression analyses (Table 3). Patients with a UACR ≥ 300 mg/g had a significantly higher risk of HCV Ab seropositivity (OR = 2.300, 95% CI 1.160‒4.562, *P* = 0.017) than patients with normal albuminuria. Although patients with a UACR of 30–299 mg/g had an increased risk of HCV Ab seropositivity compared with patients with normal albuminuria (OR = 1.463, 95% CI 0.872‒2.456, *P* = 0.150), the difference did not reach statistical significance. It is notable that the risk of HCV Ab seropositivity showed a significantly increasing trend with the severity of albuminuria (the *P* value for trend was 0.015 from normal albuminuria, moderately increased albuminuria, to severely increased albuminuria). Moreover, patients aged ≥ 65 years also had a significantly higher risk of chronic HCV infection (OR = 1.774, 95% CI 1.063‒2.959, *P* = 0.028) than those aged < 65 years, and male patients had a significantly lower risk of HCV Ab seropositivity (OR = 0.554, 95% CI 0.353‒0.868, *P* = 0.010) than female patients.

**Table 3:** Odds ratios (ORs) for HCV Ab seropositivity by the UACR and other potential risk factors using regression analyses.

Variables	Crude				Multivariate Model 1				Multivariate Model 2			
	OR	95% CI		*P*	OR	95% CI		*P*	OR	95% CI		*P*
UACR				< 0.001*				< 0.001*				0.015*
< 30 mg/g	1				1				1			
30–299 mg/g	1.741	(1.060, 2.860)		0.029	1.617	(0.979, 2.672)		0.061	1.463	(0.872, 2.456)		0.150
≥ 300 mg/g	3.195	(1.748, 5.840)		< 0.001	2.989	(1.623, 5.506)		< 0.001	2.300	(1.160, 4.562)		0.017
Age ≥ 65 years					1.829	(1.113, 3.008)		0.017	1.774	(1.063, 2.959)		0.028
Male					0.543	(0.347, 0.850)		0.007	0.554	(0.353, 0.868)		0.010
Systolic BP ≥ 140 mm Hg									1.434	(0.907, 2.268)		0.123
eGFR < 60 mL/min/1.73 m^2^									1.170	(0.709, 1.931)		0.540
ABI ≤ 0.9									0.575	(0.173, 1.915)		0.367
Use of insulin therapy									1.414	(0.849, 2.354)		0.183

*ABI* ankle-brachial index, *BP* blood pressure, *CI* confidence interval, *eGFR* estimated glomerular filtration rate, *HCV Ab* hepatitis C virus antibody, *OR* odds ratio, *UACR* urinary albumin-to-creatinine ratio.

Model 1: adjusted for age and sex; Model 2: adjusted for age, sex, systolic BP, eGFR, ABI, and use of insulin therapy.

*P value for trend.

## Discussion

The main finding of the current study was that severely increased albuminuria, but not PAD, was significantly associated with HCV Ab seropositivity in patients with type 2 DM aged ≥ 50 years. In the Third National Health and Nutrition Examination Survey (NHANES III), the prevalence of DM was twice as high in subjects who test positive for HCV antibody, and HCV Ab seropositivity was reported to be associated with an increased UACR (≥ 30 mg/g) in the subgroup without DM^19^. Similarly, Tsui et al. also reported that increased albuminuria and CKD were significantly associated with HCV Ab seropositivity, but only an increased UACR and not CKD was an independent factor for HCV Ab seropositivity after adjusting for confounding factors based on the NHANES III^20^. According to a community-based study of subjects aged 40–65 years in southern Taiwan, DM was associated with proteinuria detected using urine dipstick measurements, and HCV Ab seropositivity was significantly associated with proteinuria in the subgroup without DM^21^. The strength of the present study is that we explored the association between HCV Ab seropositivity and albuminuria in patients with type 2 DM. In particular, patients with severely increased albuminuria were at higher risk for HCV Ab seropositivity than patients with moderately increased albuminuria in comparison with patients with normal albuminuria, which emphasizes the dose-dependent association between the UACR and HCV Ab seropositivity. Kurbanova et al. also reported that HCV Ab seropositivity was associated with 50% and 95% higher odds of an increased UACR with cutoff values of 30 mg/g and 300 mg/g, respectively, in the NHANES III population, in which most individuals did not have DM^26^.

The exact mechanisms involved in proteinuria and HCV infection remain unclear. One of the possible mechanisms is that HCV infection can lead to insulin receptor defects, which induce insulin resistance^27^. Kawaguchi et al.^28^ reported that HCV promoted proteasomal degradation of insulin receptor substrates 1 and 2 via suppressor of cytokine signaling (SOCS) 3 expression in an in vitro study of transfected human hepatoma cells and an in vivo study of mice. Moreover, HCV-associated hepatic inflammation and damage may also cause insulin resistance. Konrad et al.^29^ reported that insulin sensitivity is inversely correlated with the histological activity index and fibrosis score based on liver biopsy. Narita et al.^30^ also reported that 27.5% of subjects with chronic hepatitis C infection but without known DM had abnormal glucose tolerance based on oral glucose tolerance tests, and abnormal glucose tolerance was significantly associated with the percentage of fibrosis based on liver biopsy. Shintani et al.^31^ found that in HCV transgenic mice, the ability of insulin to lower the plasma glucose level was impaired. Insulin resistance with hyperinsulinemia can contribute to renal injury through increased intrarenal production of insulin-like growth factor-1 and transforming growth factor-β and the expression of angiotensin II receptors in mesangial cells, which enhances the harmful effects of angiotensin II in the kidney. Increased endothelin-1 levels and oxidative stress and reduced nitric oxide synthesis by insulin resistance could induce renal injury^32^.

In addition to insulin resistance, antibodies induced by HCV-activating B lymphocytes may cause immune-mediated complexes, which deposit in the nephron and cause glomerular inflammation^33,34^. It is also hypothesized that HCV can directly damage the nephron because HCV particles have been found in renal tissues by electron microscopy^35,36^. Although the eGFR was lower in patients with HCV Ab seropositivity than in those without HCV Ab seropositivity, CKD was not an independent risk factor for HCV Ab seropositivity after adjusting for confounding factors in the present study. Several studies have reported that hepatitis C viral load is predictive of CKD in longitudinal follow-up^37,38^. However, according to the Taiwan National Health Insurance Research Database (NHRID), newly diagnosed HCV in patients aged ≥ 50 years did not significantly predict CKD during a mean follow-up of 7.12 years^39^. However, we did not collect data on HCV infection duration in this cross-sectional study.

Another important finding in our study was that the ABI was not significantly different between the groups with and without HCV Ab seropositivity. In line with our study, Cedarbaum et al.^40^ reported that HCV infection was not associated with PAD. In patients with regular hemodialysis, there was no significant difference in the ABI between those with or without HCV infection at baseline in an observational study^41^. The strength of our study is that a similar finding was validated in patients with type 2 DM. In contrast to the findings of the above cross-sectional studies, Sheen et al.^42^ reported that HCV was predictive of cardiovascular events in patients with type 2 DM enrolled in the P4P program. Hsu et al.^15^ reported that HCV infection was predictive of PAD according to the diagnostic codes retrieved from the Taiwan NHRID. During the 9-year cohort study, increased comorbidities were observed in patients with HCV infection, and HCV-associated comorbidities might contribute to the risk of PAD^15^. However, an ABI ≤ 0.9 had a low sensitivity in the diagnosis of PAD, especially in individuals with old age or DM^43^. The present study indicated that the ABI may have a low discriminative ability in the screening of PAD.

In the present study, HCV Ab seropositivity was associated with old age and female sex. In line with our findings, increased age and females were independent risk factors for HCV Ab seropositivity in a large Taiwanese cohort of volunteers in the Taiwan Biobank^44^. The higher prevalence of HCV Ab seropositivity in older people might result from a low seroreversion rate and persistent HCV Ab in serum that are detectable for many years^45^. Moreover, in this cross-sectional assessment, the old patients were born in the early years of a poor public health environment and had an increased risk of HCV infection^46^, such as unsterilized syringes and needles^47^. In a study using data from the Taiwan Biobank, males were reported to have a significantly lower risk for HCV Ab seropositivity than females^45^. However, the reason for increased HCV Ab seropositivity in females remains unknown.

We did not find a significant difference in ALT levels between patients with and without HCV Ab seropositivity in the present study. ALT levels could be associated with the severity of the necroinflammatory process and fibrosis, duration of chronic hepatitis, and HCV viral load^48^. Gulcan et al.^49^ reported that an increased ALT level was a risk factor for HCV Ab seropositivity in patients with DM. Korkmaz et al.^50^ also reported that ALT levels were positively correlated with the prevalence of HCV Ab seropositivity. In contrast, some studies reported that half of the patients with chronic HCV infections, even those who were untreated, displayed normal or minimally elevated serum ALT levels^51^.

There are several limitations in the present study. First, the causal relationship between HCV Ab seropositivity and albuminuria could not be established due to the cross-sectional design of the study. Second, we did not directly assess the mechanisms involved between HCV infection and the UACR in the study. Third, we did not have information on serum HCV ribonucleic acid (RNA) in the present study. Therefore, we could not assess the relationship between detectable HCV RNA and UACR or ABI in patients with HCV Ab seropositivity. The risk of kidney disease, defined as a UACR ≥ 30 mg/g or an eGFR < 60 mL/min/1.73 m^2^, has been reported to not be significantly different between resolved HCV infection (undetectable HCV RNA) and chronic HCV infection (detectable HCV RNA) in the patients with HCV Ab seropositivity; however, the risk of kidney disease might be higher in patients with HCV genotype 1 than in those with other HCV genotypes^52^. Fourth, we only included patients enrolled in the P4P program, which has been reported to attenuate chronic diabetic complications^53–55^. Fifth, we did not collect information on HCV Ab prior to the present study, and the duration of HCV infection was unknown. Finally, ABI was widely screened in patients aged ≥ 50 years in our hospital; therefore, the results cannot be applied to young patients because the prevalence of HCV infection and chronic diabetic complications are dependent on the age of the individuals in the population. It has been reported that the association between HCV Ab seropositivity and albuminuria was not significant in the population aged < 40 years^20^.

## Conclusion

In patients with type 2 DM who were aged ≥ 50 years, severely increased albuminuria, but not the ABI, was associated with HCV Ab seropositivity. Furthermore, the UACR showed a dose-dependent association with HCV Ab seropositivity. For the efficient and effective identification of potential HCV infection, screening for HCV Ab seropositivity in individuals in the above population with severely increased albuminuria is warranted.

## Methods

### Patients

Based on the clinical recommendation^25^, HCV Ab screening was performed in the outpatient department at the Division of Endocrinology and Metabolism of Taichung Veterans General Hospital (VGH) when the annual comprehensive assessment was conducted between January 2021 and March 2022 for patients who had enrolled in the diabetes P4P program but had no available HCV Ab data one year prior to enrollment. In this cross-sectional study, we retrospectively collected the medical information of patients who met the following inclusion criteria: (1) age ≥ 50 years, (2) type 2 DM, (3) enrollment in the P4P program, and (4) having undergone HCV Ab assessment in the annual comprehensive assessment between January 2021 and March 2022. We excluded patients from this study according to the following criteria: (1) no ABI data, (2) an ABI ≥ 1.4 or a history of lower-limb operation, (3) blood pressure not detected at any limb, (4) end-stage renal disease, (5) no UACR data, and (6) pregnancy. The study protocol was approved by the Institutional Review Board of the Taichung VGH in Taiwan (TCVGH-IRB No. CE22395B), with a waiver for obtaining informed consent. Anonymous demographic characteristics and laboratory data were obtained from the Clinical Informatics Research and Development Center of Taichung VGH after delinking the identification information. All methods were performed in accordance with the relevant guidelines and regulations.

### Assessment

The study data were collected from electronic medical record records, including the medical history of diabetes duration and CVD; demographic characteristics of age, sex, height, body weight, systolic BP, and diastolic BP; laboratory data of plasma glucose levels, HbA1c levels, and serum levels of total cholesterol, LDL cholesterol, HDL cholesterol, triglycerides, ALT, and creatinine; the UACR; and the ABI during the annual comprehensive assessment. According to our standard procedure in clinical practice, blood samples and urine samples were collected in the morning after an overnight fast. The current use of antidiabetic, antihypertensive, and antiplatelet drugs and statins was recorded during the annual comprehensive assessment.

HCV Ab was assayed by Elecsys Anti-HCV II (Roche Diagnostics GmbH, Mannheim, Germany). HbA1c levels were measured using cation-exchange high-performance liquid chromatography (National Glycohemoglobin Standardization Program, G8, TOSOH, Tokyo, Japan). Biochemical analyses were performed using a photometric enzymatic method with a chemical analyzer (Hitachi 7600, Tokyo, Japan). The eGFR was calculated using the modification of diet in renal disease equation as follows:

$${186} \times \left( {{\text{serum}}\;\;{\text{creatinine}}} \right)^{{ - {1}.{154}}} \times \left( {{\text{age}}} \right)^{{ - 0.{2}0{3}}} \left( { \times 0.{742}\;\;{\text{if}}\;\;{\text{female}}} \right)$$

ABI measurements were performed using a validated device (VP-1000 Plus; Omron Healthcare Co. Ltd., Kyoto, Japan). After patients had rested in a supine position for at least 5 min, cuffs that were connected to both a plethysmographic sensor for detecting volume change and an oscillometric pressure sensor for detecting blood pressure were placed on the upper arms and ankles. An ABI ≤ 0.90 was defined as PAD^14^.

Type 2 DM was clinically diagnosed by physicians, and the diagnosis was confirmed twice within 90 days before the patient was enrolled in the P4P program. CKD was defined as an eGFR < 60 mL/min/1.73 m^2^, and ESRD was defined as renal replacement therapy or an eGFR < 15 mL/min/1.73 m^2^. Poor glucose control was defined as a fasting glucose level ≥ 7.2 mmol/L (130 mg/dL) or an HbA1c level ≥ 7.0%^56^. Hypercholesterolemia was defined as a total cholesterol level ≥ 4.14 mmol/L (160 mg/dL) or an LDL level ≥ 2.59 mmol/L (100 mg/dL), and hypertriglyceridemia was defined as a triglyceride level ≥ 1.7 mmol/L (150 mg/dL) according to the reference target goals^56^. Low HDL cholesterol was defined as an HDL level < 1.0 mmol/L (40 mg/dL) in men or < 1.3 mmol/L (50 mg/dL) in women^56^. The UACR was calculated using the following formula: UACR = albumin (mg)/creatinine (g). Normal albuminuria was defined as a UACR < 30 mg/g, moderately increased albuminuria was defined as a UACR between 30 and 299 mg/g, and severely increased albuminuria was defined as a UACR ≥ 300 mg/g based on the current clinical practice recommendations from the American Diabetes Association^18^.

### Statistical analysis

All continuous data are presented as the mean ± standard deviation (SD). The categorical data are presented as the number and percentage. Independent t tests were conducted to detect significant between-group differences in continuous variables. Chi-square tests were conducted to detect differences in categorical variables. Logistic regression model which estimated OR and 95% CI was used to determine the factors associated with albuminuria and HCV Ab seropositivity. A trend test was additionally performed to examine the linear increasing trend of the risk changes associated with the UACR levels. Statistical analysis was performed using SPSS version 22.0 software (IBM Corp., Armonk, NY, USA).

## Data Availability

The datasets used and/or analyzed during the current study are available from the corresponding author on reasonable request.
